# Scaling up the task-sharing of psychological therapies: A formative study of the PEERS smartphone application for supervision and quality assurance in rural India

**DOI:** 10.1017/gmh.2024.11

**Published:** 2024-02-05

**Authors:** Daisy R. Singla, Luanna Fernandes, Katarina Savel, Ankita Shah, Ravindra Agrawal, Anant Bhan, Abhijit Nadkarni, Akshita Sharma, Azaz Khan, Anuja Lahiri, Deepak Tugnawat, Neal Lesh, John Naslund, Vikram Patel

**Affiliations:** 1 Campbell Family Mental Health Research Institute, Centre for Addiction and Mental Health, Toronto, Canada; 2 Department of Psychiatry, Temerty Faculty of Medicine, University of Toronto, Toronto, Canada; 3 Lunenfeld-Tanenbaum Research Institute, Sinai Health, Toronto, Canada; 4 Addictions and Related Research Group, Sangath, Goa, India; 5 Sangath Bhopal Hub, Bhopal, India; 6 Antarman Centre for Psychosocial Wellbeing, Panjim, Goa, India; 7 Manipal Hospital, Panaji, Goa, India; 8 Centre for Global Mental Health, Department of Population Health, London School of Hygiene & Tropical Medicine, London, UK; 9 Dimagi, Inc., Cambridge, MA, USA; 10 Department of Global Health and Social Medicine, Harvard Medical School, Boston, MA, USA; 11 Department of Global Health and Population, Harvard Chan School of Public Health, Boston, MA, USA

**Keywords:** Peer supervision, digital, measurement-based, depression, global mental health

## Abstract

Measurement-based peer supervision is one strategy to assure the quality of psychological treatments delivered by non-mental health specialist providers. In this formative study, we aimed to 1) describe the development and 2) examine the acceptability and feasibility of PEERS (Promoting Effective mental healthcare through peER Supervision)—a novel smartphone app that aims to facilitate registering and scheduling patients, collecting patient outcomes, rating therapy quality and assessing supervision quality—among frontline treatment providers delivering behavioral activation treatment for depression. The PEERS prototype was developed and tested in 2021, and version 1 was launched in 2022. To date, 215 treatment providers (98% female; ages 30–35) in Madhya Pradesh and Goa, India, have been trained to use PEERS and 65.58% have completed the supplemental, virtual PEERS course. Focus group discussions with 98 providers were examined according to four themes—training and education, app effectiveness, user experience and adherence and data privacy and safety. This yielded commonly endorsed facilitators (e.g., collaborative learning through group supervision, the convenience of consolidated patient data), barriers (e.g., difficulties with new technologies) and suggested changes (e.g., esthetic improvements, suicide risk assessment prompt). The PEERS app has the potential to scale measurement-based peer supervision to facilitate quality assurance of psychological treatments across contexts.

## Impact statement

There is a robust and growing evidence base demonstrating that frontline health care providers can effectively deliver brief psychological treatments for depression and other common mental health problems in a wide range of contexts. However, evidence-based psychological treatments remain inaccessible for the majority of the world’s population, in part due to the reliance on mental health experts to provide treatment and supervision. To overcome this challenge, frontline workers can engage in measurement-based peer supervision—a critical component to scaling-up high-quality delivery of psychological treatments. We developed the PEERS (Promoting Effective mental healthcare through peER Supervision) app—a novel, smartphone digital tool that facilitates patient registration and scheduling, and measurement-based care through session-wise data collection, rating therapy quality and assessing supervision quality. This formative research describes 1) the development of the app and 2) highlights preliminary evidence of the acceptability and feasibility of PEERS among frontline treatment providers in Madhya Pradesh and Goa, India. Through focus group discussions with frontline health care workers, we explored barriers, facilitators and suggested changes that provided valuable insight into app features and program modifications that would optimize the effectiveness of the PEERS platform. The potential implications of the PEERS app are wide reaching—it is modifiable to virtually any health care context, provider, and psychological treatment that would benefit from remote digital options for quality-assured interventions.

## Introduction

Task sharing is a proven strategy to enhance the reach of brief, psychological treatments for depression and anxiety (Van Ginneken et al., 2013; Singla et al., 2017). Worldwide, randomized controlled trials have deployed frontline non-specialist treatment providers—including community health workers, lay therapists, peers, and nurses—to enhance the reach of effective psychological treatments. Supervision is a critical component of this approach, both to support providers and to assure the quality of psychological treatments. Specifically, supervision is a key pedagogical and quality assurance tool in treatment delivery, which is known to positively impact the therapist in training (Saxon et al. 2016; Fairburn et al., 2017; Kühne et al., 2019), therapy quality (Weck et al., 2016; Kühne et al., 2019) and patient outcomes (Kühne et al., 2019; Saxon et al., 2017; Singla et al., 2020; 2023).

A dependence on mental health specialists to supervise providers in-person is not scalable. Specialists are not readily available and in-person methods are expensive and time-intensive (Fairburn and Patel, 2017). This is especially true in low-resource settings where there are few mental health specialists who are adequately trained in psychological treatments and available to provide supervision (Singh, 2018; Mondal et al., 2021), and where existing frontline providers face enormous competing pressures on their time related to other health care tasks, impeding their ability to attend in-person supervision. A lack of scalable and quantifiable methods of supervision in real-world settings also represents a major barrier to the sustainability of the quality of the delivery of psychological treatments. The current study seeks to address these barriers posed by the orthodox approach to supervision.

Peer supervision (in contrast to the usual approach of expert supervision) occurs when therapists with similar levels of training, monitor, evaluate and support one another (Akhurst and Kelly, 2006; Golia and McGovern, 2013). Peer supervision relationships are built around mutual responsibility, accountability and a desire for improvement (Spence et al., 2001). Peer supervision also provides an opportunity to engage in self-care (Schumann et al., 2020), draw upon each other’s experiences (Golia and McGovern, 2013) and take active roles in assisting one another to alleviate stress (Yeh et al., 2008; Schumann et al., 2020). Measurement-based peer supervision (MBPS) is a novel innovation that aligns with the established quality improvement strategy of ‘measurement-based care’. MBPS offers a quantitative compass to monitor and improve the quality of the delivery of psychological treatments through objective, measurable metrics that can be compared across providers and over time. Based on social cognitive learning theory (Bandura, 1986), peer supervision has the potential to enhance social support between peers and facilitate peer learning. This strategy involves the use of psychometrically-sound rating scales to assess the quality of audio-recorded therapy sessions. Ratings by the treatment provider (self rating), peer counselors (average peer rating), and a clinical expert (expert rating) are conducted prior to and then shared during the peer supervision session. The review of the ratings is complemented by a group discussion and narrative comments.

Our previous work in India has demonstrated the acceptability, feasibility and efficacy of MBPS models among lay and peer providers in two large randomized controlled trials using brief psychological treatments for depression (Patel et al., 2017; Fuhr et al., 2019). We found that providers perceived peer groups to be empowering and that they were able to assess the therapy quality of audio-recorded sessions using rating scales as reliably as expert supervisors (Singla et al., 2019; Singla et al., 2014). We also found that peer supervision in a group format served as a supportive space for problem-solving around common challenges (Singla and Kumbakumba, 2015; Singla et al., 2019).

In addition to peer-led supervision, virtual supervision through a mobile device is one solution to increase access to quality-assured supervision. For example, a recent study in Kenya found that using mobile phones for supervision was generally acceptable and feasible as reported by lay providers delivering trauma-focused cognitive behavioral therapy for trauma (Triplett et al., 2023). Notable facilitators reported by lay providers included the ease and convenience of virtual supervision (e.g., ease of reaching their supervisor) and digital literacy (Triplett et al., 2023). However, to the best of our knowledge, no study has used measurement-based metrics (described above) to facilitate quality-assured supervision in low-resource settings. It is critical to answer these questions using methods in research co-design (Slattery et al., 2020) and human-centered design (Black et al., 2023) to ensure the proposed solution is both acceptable and feasible for the target user group—in this case, frontline treatment providers.

The overall aim of PEERS (Promoting Effective mental healthcare through peER Supervision) is to facilitate quality-assured therapy among frontline providers delivering psychological treatments. In this study, we intend to 1) describe the development and 2) assess the acceptability and feasibility of a novel digital tool (PEERS) for remote MBPS, an innovation we have designed, piloted and evaluated over the past decade (e.g., Singla et al., 2014, 2019), using one of the most widely used digital platforms for supporting frontline provider-delivered care in global health. We outline the process and outcome of app development, modification and input, including conducting training and orientation among frontline treatment providers, and garnering qualitative inputs from providers and clinical experts.

## Methods

### Setting and participants

**Figure 1.**
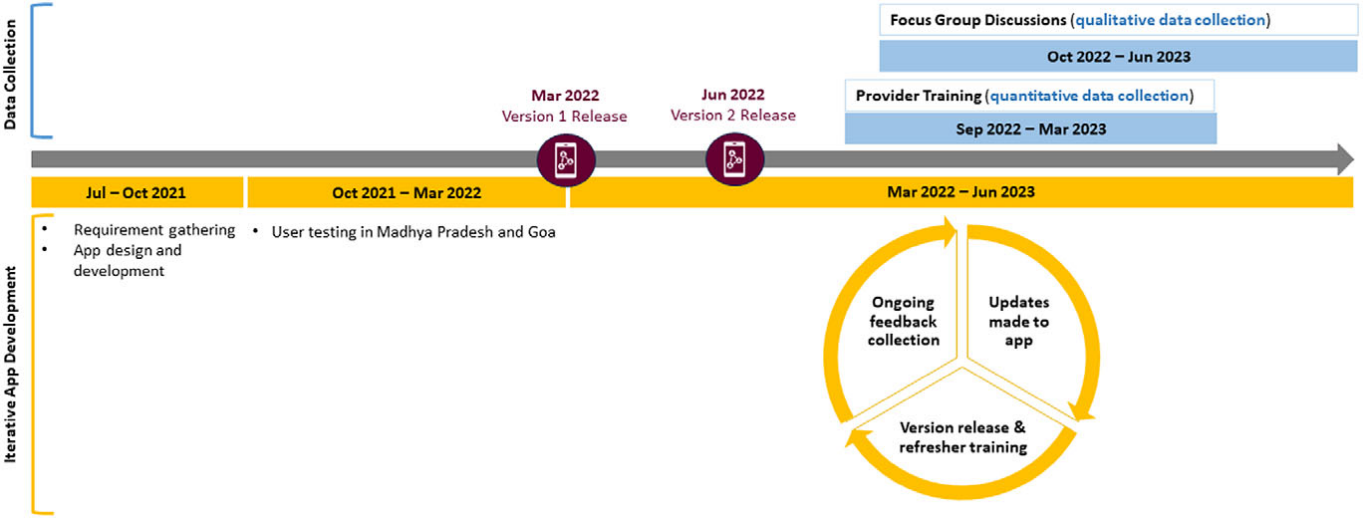
Time course of data collection and the iterative PEERS app development process.

The current study is embedded in two programs to scale up the Healthy Activity Program (HAP) in two states in western and central India, Goa and Madhya Pradesh, respectively. HAP is a brief, manualized behavioral activation treatment (Chowdhary et al., 2016) that has been shown to improve symptoms of moderately severe to severe depression when delivered by lay counselors in primary healthcare settings (Patel et al., 2017). The strategies in HAP include psychoeducation, behavioral assessment, activity monitoring, activity scheduling and problem-solving. HAP is typically delivered in an individual format over six to eight sessions and on a weekly to fortnightly basis. Each frontline provider in these two programs is requested to carry a caseload of 2–4 patients at a time, given their other responsibilities. For the current study, most providers had not started delivering treatment. The data were collected between August 2021 and March 2023 during the development and post-deployment phase of the PEERS app version 1 (launched in March 2022) and version 2 (launched in June 2022) (Figure 1). Ethical approvals were obtained through Sangath (EMPOWER: RA_2023_90; IMPRESS: AN_2017_033), Sinai Health (CTO 3614) and Harvard Medical School (IRB21-0992).

#### Goa

In Goa, the current study is embedded in the IMPlementation of evidence-based facility and community interventions to reduce treatment gap for depRESSion (IMPRESS) trial. IMPRESS aims to scale up HAP through the existing primary health care system. Over the course of five face-to-face trainings (Sep 2022–Feb 2023), IMPRESS trained a range of non-specialist providers deployed in public primary health care facilities (including nurse midwives, medical officers, multi-purpose health workers, counselors and staff nurses) in the HAP treatment, the PEERS app, and supervision procedures. Providers were trained and supervised by experienced clinical experts who were credentialed to become HAP counselors and had achieved a minimum score of 3 on the EMPOWERS scale for supervision quality (described below). Clinical experts have post-graduate qualifications, a profound understanding of the local context, and have successfully completed comprehensive HAP training, including a rigorous internship program.

#### Madhya Pradesh

In Madhya Pradesh, the EMPOWER program is training community health workers known as Accredited Social Health Activists (ASHAs) to address the treatment gap for depression (Patel et al., 2022). ASHAs are exclusively female residents of the villages they serve and are deployed at a ratio of approximately one ASHA per 1,000 population. EMPOWER ASHAs were previously trained in HAP between March 2021 and 2022 and had begun the process of identifying patients with depression in the community, delivering HAP, and being supervised by clinical experts. Over the course of six face-to-face trainings (Oct 2022–Jan 2023), ASHAs from one EMPOWER district (Narmadapuram) were invited to be part of the current study, followed by participation in the PEERS app orientation.

#### Supervision

Two types of supervision were used in the current study to ensure quality delivery of HAP. In the PEERS digital platform, the supervision type can be identified to differentiate between the two types of supervision:

1. *Measurement-Based Peer Supervision (MBPS).* Based on established procedures (Singla et al., 2014, 2019), MBPS provides quantitative measures to monitor and improve treatment delivery, by having treatment providers, their peers and a clinical expert listen to a selected audio recording of a treatment session and then rate the therapy quality. Accordingly, the PEERS app incorporates the Behavioral Activation Quality Scale (BAQ-S; Singla et al., 2022, Supplementary Figure S1; adapted from the Q-HAP, Singla et al., 2014), which assesses therapy quality using two domains: ‘treatment-specific skills’ specific to behavioral activation; and ‘general skills’ that cut across treatment modalities (e.g., collaboration, empathy). Each domain-specific subscale is rated on 10 items, using a mean score ranging from 0 (inadequate) to 4 (excellent). In this type of group supervision, each treatment provider listens to and rates audio recordings of the selected treatment session and provides constructive feedback to peers in their group through the app. All treatment sessions are recorded on the PEERS app, and clinical experts randomly select one treatment audio per MBPS session from this pool of audio recordings. Through this process, approximately 30 to 50% of treatment sessions are typically rated in either context. Rating of a session typically takes 30 to 60 minutes to complete, depending on the length of the session and experience level of the provider (newer providers generally take longer to rate treatment sessions). Providers are given 1 week to complete the rating to allow for flexibility in completing this task amidst other professional responsibilities. Following this, providers attend primarily virtual group supervision meetings bi-weekly to monthly to discuss the ratings and any other issues that are proposed by the providers. In addition, treatment providers meet bi-weekly in a group to roleplay clinical scenarios to improve counseling skills in handling difficult cases; there is no measurement of therapy quality.
2. *Individual Supervision.* Individual supervision is a form of one-to-one supervision held between the provider and their clinical expert which focuses on an individual provider’s caseload and skill development. Individual supervision is scheduled monthly in Goa, and as required in Madhya Pradesh based on the needs of individual treatment providers.

### Development of the PEERS app

The PEERS app was developed in collaboration with Dimagi—a pioneering technology firm that has built the open-source CommCare platform of mobile technologies to support frontline health workers in low-resource settings globally (Labrique et al., 2018). For the current study, the PEERS digital tool was built on CommCare and tailored for the delivery of HAP by frontline treatment providers, and engagement in MBPS.

The study team provided the content and workflows of the app based on evidence-informed procedures of the HAP treatment, measurement-based care and supervision. The app development process included three pivotal phases (Figure 1): (1) *Requirement gathering*: This involved defining workflows and collecting essential data points related to the HAP treatment and MBPS; (2) *Design and development*: Adopting an iterative approach, this phase included constructing wireframes, integrating workflow questionnaires, and incorporating Hindi (local language) content; and (3) *Internal testing*: Before beta and user testing, the app underwent rigorous quality assurance to detect and address potential issues and updates.

Once the content was established, the process of beta testing the app involved two phases, each serving a specific purpose to ensure the application’s quality and usability: (1) *Phase 1: Internal Quality Assurance Testing:* Before releasing the application version, the Dimagi Quality Assurance team conducted extensive testing on all application workflows. This internal quality assurance testing helped identify and address any issues or bugs before proceeding to the next phase and final release; (2) *Phase 2: Testing with Users:* We employed an interactive process by conducting three rounds of user-level testing with ASHAs in Madhya Pradesh. These users were selected as they represented both smartphone-experienced and non-smartphone-experienced providers, and providers who had previously received HAP training. Through testing sessions conducted between October 2021 and March 2022, we collected valuable user feedback from the interactions. Activities during testing sessions included: (1) App installation and setup testing on different smartphones; (2) functional testing by simulating real-world scenarios while engaging with the app; and (3) collecting user feedback through focus group discussions and one-on-one interactions. With each testing session, we focused on comfort with using a smartphone, and technical and functional workflow understanding. While beta testing occurred in controlled environments, it reflected real-world personnel using these features among real patients in pragmatic primary health care and community settings.

Inputs from treatment providers, clinical experts and the research team resulted in the addition of new features and updates to existing workflows. Subsequent iterations focused on refining the workflows within these existing applications. At each version release, relevant trainings and demonstrations were provided to ensure a smooth transition and understanding of the updated workflows. This included treatment providers having the opportunity to practice using the app on “demo mode” using mock data, as opposed to real mode through which patient and supervision data is collected and uploaded to the CommCare server.

### Aims and components of the PEERS app

**Figure 2.**
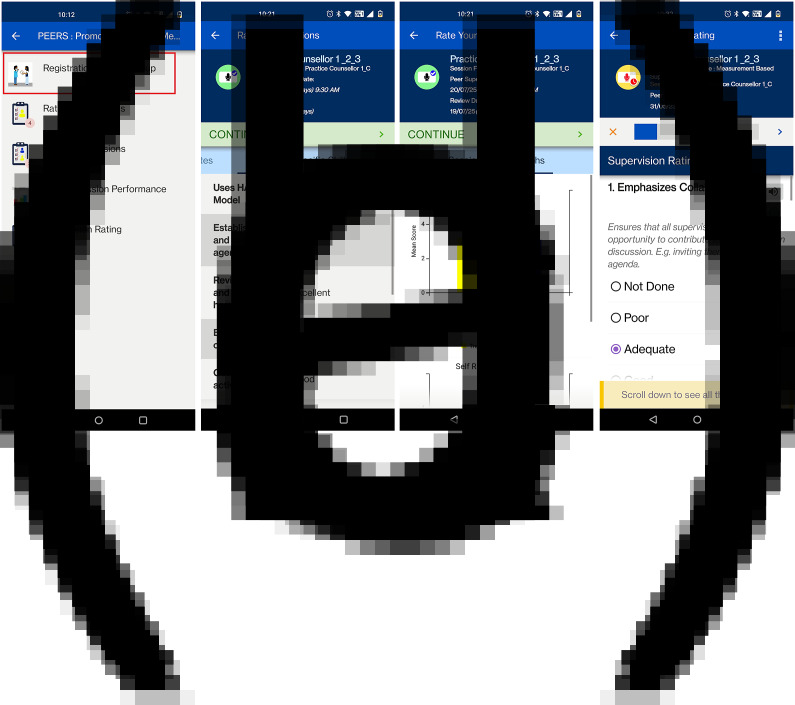
Visuals of the PEERS app: (a) start menu screen; (b) BAQ-S scale rating; (c) mean BAQ-S self-rating; (d) EMPOWERS rating scale (question 1).

The resultant PEERS app (Figure 2) has three overarching aims: (1) registering and scheduling patients, (2) collection of session-wise data including symptom scores and session notes and (3) MBPS. Registration and data collection functions include collection of demographic information, collection of session-specific details (e.g., session location), scheduling HAP sessions, audio recording of treatment sessions, checklists to guide treatment delivery, and reasons for treatment closure and patient discharge. Supervision-specific functions include sharing treatment session audio recordings for MBPS, accessing and rating treatment session audio recordings, and rating clinical experts and peer supervisors on the quality of the delivered supervision using the 8-item EMPOWERS supervisor rating scale (Supplementary Figure S1). EMPOWERS ratings are completed by both the participating treatment providers and the supervision facilitator following each group supervision session. Supervision-specific skills such as organization, collaboration, and being non-judgemental are rated on a scale ranging from 0 (inadequate) to 4 (excellent) to produce a mean EMPOWERS score for both group- and self-ratings.

Apart from the PEERS app which could be installed on the providers’ smartphones, two related applications were designed. First, the *Supervisor Web Application* was designed to allow clinical experts to oversee MBPS and support providers. Key functions include supervision scheduling, tracking individual provider performance and aggregate group supervision metrics (e.g., attendance, average EMPOWERS ratings), and sharing required outputs for MBPS (e.g., EMPOWERS and BAQ-S ratings). Second, the *PEERS E-* *Course* is a digital guide for providers to learn how to use the PEERS app. The course is hosted on online learning management platforms and can be accessed via a web browser or an Android smartphone app, and is designed to supplement the initial PEERS training (described below). The course has five modules through which learners understand the concept of supervision, types of supervision, the skills for conducting supervision, the features of the PEERS app, and strategies for self-care. Multiple-choice quizzes follow each module to evaluate the providers’ understanding of the material. E-Course progress and quiz scores are captured through the learning management platforms that host the E-Course.

### PEERS orientation and training

Orientation to and training on the PEERS app and supervision procedures were delivered over a 1-day, in-person session. The key topics covered were understanding supervision, learning why supervision is important, and using the PEERS app to register and schedule patients, conduct a treatment session, listen to session audio recordings, and submit ratings on the BAQ-S and EMPOWERS scales. Orientations were led by clinical experts at each site and supplemented by the PEERS E-Course. Clinical Experts were trained on the PEERS platform and supervision procedures in two, 1-hour trainings which included orientation to the PEERS app and supervisor web app (facilitated by Dimagi) and orientation to supervisor processes by the PEERS research team.

### Data collection and analyses

Quantitative data collection took place from September 2022 to March 2023 (Figure 1). Quantitative measures include descriptive statistics (mean scores and standard deviations or frequencies where relevant) related to the number of providers recruited and trained, their demographic characteristics (i.e., age, gender, occupation, and history of mental healthcare training and delivery), and their training progress (i.e., completion of PEERS orientation and E-Course). Treatment provider demographics were collected and entered on an online REDCap™ survey by independent trained research assistants. Training and E-Course progress were captured through the mentioned learning management platforms.

Qualitative data collection took place from October 2022 to June 2023 through focus group discussions (FGDs) following orientations with available treatment providers and clinical experts (Figure 1). Data collectors followed semi-structured topic guides focused on key themes pertaining to the feasibility and acceptability of PEERS. This included questions about barriers and facilitators to training and to using the app and suggested improvements. FGDs were conducted in the preferred languages of the providers (English, Hindi and Konkani) and were transcribed and translated into English for analysis. FGDs were approximately 2 hours in length and were recorded with consent using handheld recording devices.

Data were analyzed using a thematic analysis approach (Ritchie and Spencer, 2002), which includes a five-step process to analysis: (1) familiarization with the data; (2) identification of a thematic framework to examine relevant barriers, facilitators and suggested modifications; (3) indexing (coding raw data); (4) charting and finally; (5) interpretation. In this case, our thematic framework adapted the standards for mental health app quality control recommended by Torous et al. (2019) to examine the following themes from the perspective of treatment providers and/or clinical experts: (1) *Training, orientation and education on the PEERS platform*—users’ experience of learning how to use the app, deliver treatment and engage in supervision; (2) *app effectiveness*—users’ perspectives on the efficacy and utility of the app per its target functions; (3) *user experience and adherence*—users’ perspectives on the usability of the app and experience in supervision as it relates to day-to-day integration and uptake within the clinical practice; and (4) *data safety and privacy*—users’ perspectives on patient and data confidentiality as it relates to the app. A minimum of two researchers in both contexts (BB and AJ in Goa, and AL, SM, AS, LS and NJ in Madhya Pradesh) carried out the qualitative analyses. The transcribed data for each interview was read and reread to gain familiarity with the raw data. During this process of familiarization, the emerging codes were highlighted. These emerging codes were compared to identify themes in the raw data. A coding index (also known as a thematic table) was developed to organize the emerging codes into themes and sub-themes. The researchers then met with the rest of the research team at their respective sites to agree upon a common thematic framework, and re-coding was carried out where necessary. The data was then translated and independently coded. Once interviews were coded, charting was conducted to categorize the data according to their appropriate theme and sub-theme. Frequencies for each code were eventually calculated and ranked according to their frequency by setting. These rankings were then used to compare results from the two sites.

## Results

Step 1: ***Training and orientation of providers to the PEERS platform.***

Table 1 includes details on provider demographics and training progress. Most (93%) treatment providers were female, were between 30 and 35 years of age, and the majority (61.37%) had no previous experience of delivering any mental healthcare. Most (65.58%) completed the digital PEERS E-Course which took, on average, 2.5 h to complete.

Step 2: ***Garner user feedback from participants and clinical experts.***

Following the initial version 1 release in March 2022, we collected feedback from providers to assess their experience with PEERS training and use of the PEERS app after training. Eleven focus group discussions were conducted across sites with a total of N = 98 participants. In Madhya Pradesh, 8 clinical experts were interviewed in one FGD [mean age 39.87 years (SD = 5.01), 63% female] and 26 frontline providers were interviewed across 3 FGDs [mean age 34.42 years (SD = 5.74), 100% female]. In Goa, 6 clinical experts were interviewed in one FGD [mean age 34.50 years (SD = 7.40), 83% female], and 61 frontline providers were interviewed across 5 FGDs [mean age 33.16 years (SD = 7.01), 77% female].

**Table 1.** Training progress and demographics

	Madhya Pradesh	Goa
Training progress		
Total trainees	144	71
Number of training conducted (date range)	6 (Oct 2022 – Jan 2023)	5 (Sep 2022 – Feb 2023)
Trainee demographics		Only available for 44 trainees in Goa
Age (years)		
Mean (SD)	32.42 (5.74)	33.16 (7.01)
Range	23 to 49	22 to 55
Percent female	100%	80.28% (57/71)
Years of experience in healthcare service (mean SD)	—	7.48 (6.03)
n (%) with previous mental healthcare training	—	18 (40.91%)
n (%) with previous mental healthcare experience	—	17 (38.63%)
E-Course progress		
Completed E-Course (n (%))	97 (67.36%)	44 (61.97%)
Quiz scores		
Mean (SD)	24 (10.12)	15.95 (2.40)
Score range^a^	0–36 (scale range 0–36)	10–18 (scale range 0–18)

a Due to the use of two different learning platforms, the E-Course quiz scale range is 0–36 in Madhya Pradesh (2 points allocated for each correct response) and 0–18 in Goa (1 point allocated for each correct response).

Key themes endorsed across sites are summarized in Table 2. In general, most treatment providers and clinical experts reported encountering more facilitators than barriers when using the app, indicating the majority of responses for key themes related to acceptability (user experience and adherence) and feasibility (app effectiveness). The main themes are summarized in the sections that follow.

Theme 1: ***Training, Orientation and Education***. Facilitators to training and education included training provided in the local language, sufficient technological support, guidance on managing burnout, and training modality. Providers also reported that support from peers, clinical experts, and the research team was facilitative to their learning. For example:

- *“It was very simple, they explained it very well, and she did a role play so that we could understand it better.” (FGD4,P4, Madhya Pradesh)*

**Table 2.** Barriers and facilitators of the PEERS program

Site	Madhya Pradesh		Goa		Suggested changes across sites
Sample	n = 26 treatment providers; n = 8 clinical experts		n = 61 treatment providers; n = 6 clinical experts		
Key theme	Barriers	Facilitators	Barriers	Facilitators	
Training, orientation and education	Difficulty with PHQ-9 scoring (++) Lack of time to practice using the app (++) Understanding the app interface (+)	Knowledge gained in using the app, PHQ-9 scoring, identifying depression and conducting sessions (++) Liked the training and eagerness to learn new things (+) Training in Hindi (+) Rigorous practice (+) **Use of role plays** (+) Support by clinical experts and peers (+)	Bugs in app demo version (++) Navigating the app in demo versus real mode (++)	Ease of use of the app (+++) Topics covered on work/life balance and managing burnout (+++) Tech assistance by the research team (+++) Knowledge gained in using the app (++) **Use of role plays** (++) **Support and feedback by clinical expert** (++)	Provider suggestions Overview of the full app at the start of training (++) Addition of reference videos and documents for app processes (++)
					Clinical expert suggestions Differentiating between demo and real mode (++)
App effectiveness	PHQ-9 not visible in the app while recording a session (++) Inconsistent audio recording (+)	**Ease of record keeping (+)** **Self- and clinical expert feedback with recorded sessions (+)**	No feature for additional, ad hoc clinical notes (++) Potential for tech issues before sessions (++)	Ease of use of the app (+++) **Consolidated patient data (++)** Comprehensive documentation and session guidance (++) **Comprehensive feedback on therapy quality (+)**	Provider suggestions Emphasizing time period of PHQ-9 questions (+)
					Clinical expert suggestions Suicide risk assessment pop-up per patient scores (+++) Follow-up questions specified per PHQ-9 scores (+++) Resolve issues with audio recording sessions (+++)
User experience and adherence	Network issues (++) Poor quality mobile (++) Lack of focus during supervision due to number of patients to discuss (+) Poor battery life (+)	Peer teamwork contributing to higher quality of care (+) Increased ease of finding and helping patients (+) Prompt supervision and **scheduling per treatment providers’ schedules (+)**	Increased screen time (++) Scheduling challenges for group supervision (++) Potential connection issues with virtual supervision (++)	Enjoyment of monthly, in-person supervision (++) **Knowing the schedule** and agenda for supervision in advance (++)	Provider suggestions More frequent in-person group discussion (+) Extend scheduling options for the next session past 15 days from the current date (+)
					Clinical expert suggestions Change color of buttons for user experience (++)
Data safety and Privacy	Potential hesitation among patients to share information with treatment providers from their same village (++)		Potential for patients to not be forthcoming when sessions are recorded (+)	Ease of accessing patient data, including PHQ history and homework (++)	

Note: ‘+’ some participants endorsed this theme (x < 20%); ‘++’ a good portion of participants endorsed this theme (21% < x > 59%); ‘+++’ wide majority (x > 60%) of participants endorsed this theme. Bolded themes are shared across sites.

Providers also expressed that training contributed to increased knowledge related to identifying depressive symptoms using the Patient Health Questionnaire-9 (Kroenke et al., 2001)—the depressive symptom measure used in the PEERS app to conduct HAP sessions and attend supervision. For example:

- *“About the app… We get to know in detail about how many sessions we have done, what we should do, what activities should be given to them, summary. Everything is there. So, that app is good for us. It is good for us to remember as well. Like when is the next follow up, who will come (and) when. It is good as it reminds us.” (FDG1_NG, P1, Goa)*

In contrast, barriers to training were related to technological difficulties with early adoption of the app—such as PHQ-9 scoring and toggling between demo and real mode—as well as programmatic challenges. For example:

- *“I had a difficult time in recording the correct score for questions. I also had trouble in remembering those questions.” (FGD4, P4, Madhya Pradesh)*
- *“It was not clear on day of training as well. (The trainer) had also explained to us twice but it could not sit in our mind at that time and went over the head. The session can be recorded…at first, I was not able to comprehend it.” (FGD4, P3, Madhya Pradesh).*

Clinical experts also reported that providers struggled with differentiating between various modes of the app, as well as with updating the app and remembering to rate sessions. Accordingly, suggested changes by clinical experts were related to improving user experience and ease of conducting sessions. For example, one expert described:

- *“First for learning you have to do it in practice mode and when you do it in real then you should login using password, this is your password and then this is your real patient data and you have to submit it. You must go here, and even after being told several times; they remain perplexed” (FGD1, P5, Madhya Pradesh)*

Suggested modifications to training were related to training flow and modality, demonstrating use of the PEERS app, and providing additional supplementary information. For example:

- *“From time to time, like in training, group discussions should also be held with us in between.” (FGD2, P1, Madhya Pradesh)*
- *“We did the full process yesterday… from the start till the end. So, if suppose we had seen it in the beginning, of what should be there in each part, so that would be better." (FDG1_SN, P2, Goa)*

Theme 2: ***App Effectiveness***. Key facilitators reported were also related to the clinical utility of the app and ease of digital record-keeping. For example, most treatment providers noted the convenience of accessible and consolidated patient data—for example, PHQ-9 history and homework:

- *“I really appreciate the convenience that the app provides… it stores all the details in the mobile and this data can be accessed anytime, anywhere.” (FGD4, P10, Madhya Pradesh)*

Barriers to app effectiveness included challenges related to specific capabilities including the lack of a feature for recording ad-hoc clinical notes, inconsistent audio recording during treatment sessions, and PHQ-9 scores not being visible and available for reference while recording a session. Reported facilitators were ease of use of the app, and receipt of comprehensive feedback and support from the clinical expert on therapy quality. For example:

- *“We will not be alone…our supervisors will help us. Or they will explain us by doing role plays. This is one belief that we have." (FDG1_NG, P3, Goa)*

Suggestions from clinical experts to improve effectiveness included an automatic prompt to conduct a suicide risk assessment per the patient’s self-reported PHQ-9 scores, follow-up questions specified per PHQ-9 scores and resolving issues with audio recording treatment sessions.

Theme 3: ***User Experience***. Facilitator’s to experience and adherence included flexible scheduling of supervision sessions, and knowing the peer group meeting schedule and agenda in advance. For example:

- *“(Our clinical expert) used to call and ask are you free…we ourselves had told the time of 3:00 pm, then they used to help us to practice by becoming patients.” (FGD2, P1, Madhya Pradesh)*

Providers also reported on the benefits of collaborative learning through group supervision. For example:

- *“This is also very good work. (The study coordinator) did a great job by connecting us with each other… we benefited even better, as they formed a group by joining two people.” (FGD2, P3, Madhya Pradesh)*

Barriers to user experience and adherence included programmatic and implementation-related concerns such as potential network issues and difficulties scheduling supervision sessions. For example, one treatment provider described:

- *“(If) there is network issue…it runs very slow and sometimes it doesn’t work at all.” (FGD4, P8, Madhya Pradesh)*

Clinical experts also reported that treatment providers faced difficulties with updating the app when a new version was released, remembering to rate treatment and supervision sessions on therapy and supervision quality, respectively, and that they lacked general experience with group conference calls and using smartphones.

Suggestions to improve experience by treatment providers included extended scheduling options for treatment sessions (e.g., when the next session is more than 3-weeks away) and more frequent in-person group meetings. Clinical experts also suggested esthetic changes to the app to improve user experience—in this case, changing the color of buttons within the app.

Theme 4: ***Data Safety and Privacy***.

Treatment providers shared concerns related to confidentiality. These included potential hesitations among patients to share information with providers from their same village and potential for patients to not be forthcoming when sessions are recorded:

- *“Still, we have this that when the patient will talk, it will remain in his mind that there is recording so he might uh -- there will be more chances that he will keep thing within him and talk, because of the recording.” (FDG2_SG, P2, Goa)*

Step 3: **Modifications to the App based on User Experiences.**

During the course of app development and beta testing, a number of changes were made to the PEERS app and supervisor web application based on the results of field testing and ongoing collection of user feedback. Key modifications are summarized in Table 3.

**Table 3.** Modifications made to the PEERS app and supervisor dashboard

Function Category	Description
PEERS app	
Cosmetic	Updated icons to enhance the visual experience within the application
Training	Addition of four demo HAP recordings have been added to the practice version of the application to provide users with examples of how sessions can be recorded and rated in the app
Patient Flow	Mandatory collection of the Patient Identification Number during patient registration Addition a of a treatment phase checklist to guide HAP delivery Addition of a suicide risk assessment summary Mechanism to discharge patients from treatment based on clinical criteria (PHQ-9 < 5) Restriction of PAAS scale questions to be displayed from the second session onwards, as completion of the PAAS requires the therapist to have knowledge the patient’s engagement post-session one Addition of the “Record Patient Outcome” form to enable the recording of patient outcomes without conducting a new HAP session
Data validation	Validation check for Patient Identification Number to prevent user errors during registration Additional labels and confirmation checkboxes to assist counselors in submitting proper data and completed forms
Supervision	Addition of supervision management workflows for treatment providers who graduated to the role of HAP Peer Supervisor Automatic review reminders for providers in advance of peer supervision
Supervisor dashboard web application	
Supervision	Addition of average self-, peer- and supervised-rated Q-HAP scores displayed in each treatment provider’s case details, with the option to filter by completed measurement-based sessions Addition of attendance function to track treatment provider participation in peer supervision Addition of a scheduling function for measurement-based, non-measurement-based, and individual supervision sessions for up to 30 days from the current date to allow for more flexibility and efficient planning of supervision sessions

## Discussion

The current study presents formative research that describes the development and preliminary acceptability and feasibility of the PEERS platform—a novel, digital tool to facilitate the delivery and supervision of non-mental health specialist providers scaling up a brief behavioral activation treatment in two states in India. Key findings are discussed below.

First, due to the rigorous process of developing and testing with key stakeholders, the PEERS digital tool experienced multiple modifications involving the utility and cosmetic appearance of the app and dashboard to improve user workflow and experience. Similar to others focusing on a co-design process with users (Triplett et al., 2023), inputs came from varied stakeholders ranging from study team members with extensive clinical experience to non-specialist providers with minimal or no previous training in mental healthcare. One strength of the study was the collection of data from numerous key users—namely the non-specialist treatment providers delivering a brief, evidence-based psychological treatment in real-world settings and their clinical experts. This is important because treatment providers are typically not the main source when designing, developing and reviewing health apps (Schueller et al., 2018), which may compromise app clinical utility, effectiveness and safety (Lagan et al., 2020), and potential for uptake and use. Benefits of leveraging the voices of those in health care and community settings inform the clinical utility, safety, validity and feasibility and acceptability of health apps and this may be especially important in the global mental health context (Chan et al., 2017). This helps to ensure that apps are *culturally accessible*—i.e., whether the app works effectively in the user’s culture—in this case, by lay counselors delivering low-intensity interventions in primary care settings.

Second, we have identified two distinct groups of key facilitators: those non-specific to the app and those specific to the app. Those that are non-specific to the app included both providers and clinical experts endorsing interactive elements such as role plays, peer support and receiving feedback. These elements are not unique to the PEERS platform. Interactive teaching elements and feedback are often reported as facilitators by trainees—and irrespective of their previous background in mental healthcare and whether or not technology is used (e.g., Borders, 2012; Singla and Kumbakumba, 2015; Kühne et al., 2019). The second group of facilitators reported by treatment providers were consolidated patient data, recording keeping and scheduling of treatment and supervision sessions. These features promoting the clinical utility of the PEERS app may ultimately improve therapy quality by helping the treatment provider and clinical expert be more organized, and are generalizable to the delivery and supervision of any psychological treatment supported by the PEERS app.

Finally, common barriers reported were related to the use of technology itself. Some treatment providers reported increased screen time, network and connectivity issues and adjusting to the features of the app including using the various modes of the app. As a result, multiple modifications were required to facilitate provider workflows and experience, while considering contextual factors such as novelty of the app and supervision procedures. Similar barriers were reported in an evaluation of the feasibility and acceptability of a digital HAP training program application delivered on tablets, mobile devices, and laptops, among community health workers (in this case, ASHAs) in rural India (Muke et al., 2019; Muke et al., 2020). These included low familiarity with device features and how to use the device, poor audio quality, and slow internet; app-related challenges included content loading improperly, bugs, and difficulties navigating the app interface. These challenges are expected with the adoption of new technology in novel settings and may be addressed by prioritizing engagement and motivation among users, as well as providing additional training and appropriate support from qualified technical staff (Labrique et al., 2018). For example, all treatment providers undertook a validated, digital orientation as part of their HAP training—informed by pilot work in comparable settings (Muke et al., 2020)—and received appropriate support to facilitate training completion.

Limitations of the study include convenience sampling for qualitative data. Our study sample was based on available treatment providers who had undergone training and orientation to the PEERS platform, rather than those who were randomly selected. Random sampling of treatment providers may ensure all perspectives are being represented. Ongoing data will be collected to examine quantitative statistics of user experience, and facilitate future mixed methods studies using both quantitative and qualitative perspectives. As the current study continues, we aim to continue to examine user experience, along with whether PEERS app will be used by various non-specialist treatment provider cadres to facilitate improved therapy quality and in turn, patient outcomes in two different contexts in India. Additionally, all treatment providers in Goa and a small subset in Madhya Pradesh were provided with smartphones to ensure devices met necessary technological specifications. Access to a smartphone that can support the PEERS app may be a barrier to widespread uptake and scalability of the app. While smartphone ownership is increasing in the population, health care systems are increasingly providing smartphones to frontline staff. Thus, this practice reflects the pragmatic setting.

Importantly, our mixed-methods evaluation of the PEERS app is ongoing. While the current study focused on the development of the app and supervision protocols, future work will report on the implementation of the app in varying contexts in Goa and Madhya Pradesh, India. Further questions will examine the feasibility, acceptability and cost of the app as it relates to treatment delivery, the potential relations between therapy quality scores and patient outcomes, provider perspectives on peer-led measurement-based supervision, and the utility of the PEERS E-Course as a tool for providing follow-up support and improving scalability and accessibility of the in-person PEERS training, among others.

To our knowledge, this is among the first studies to document the development and formative research of a mental health digital app to facilitate quality-assured psychological treatments in low-resource contexts. Both treatment providers and clinical experts across sites reported multiple and important facilitators, barriers and suggestions to inform key areas to improve the PEERS app in areas of utility, adherence and effectiveness. The goals of the ongoing current study will inform whether this app will be scaled to facilitate quality-assured psychological treatment for depression in two varying contexts across India, with prospective applications to a range of treatments and contexts worldwide.

## Supporting information

Singla et al. supplementary material Singla et al. supplementary material

## Supplementary material

The supplementary material for this article can be found at http://doi.org/10.1017/gmh.2024.11.
