# Scaling up the task-sharing of psychological therapies: A formative study of the PEERS smartphone application for supervision and quality assurance in rural India – CORRIGENDUM

**DOI:** 10.1017/gmh.2024.61

**Published:** 2024-05-30

**Authors:** Daisy R. Singla, Luanna Fernandes, Katarina Savel, Ankita Shah, Ravindra Agrawal, Anant Bhan, Abhijit Nadkarni, Akshita Sharma, Azaz Khan, Anuja Lahiri, Deepak Tugnawat, Neal Lesh, Vikram Patel, John Naslund

**Keywords:** Peer supervision, digital, measurement-based, depression, global mental health

The order of the author list on the paper was incorrectly listed with the last 2 authors the wrong way round:

Daisy R. Singla, Luanna Fernandes, Katarina Savel, Ankita Shah, Ravindra Agrawal, Anant Bhan, Abhijit Nadkarni, Akshita Sharma, Azaz Khan, Anuja Lahiri, Deepak Tugnawat, Neal Lesh, John Naslund and Vikram Patel

This should have been listed as:

Daisy R. Singla, Luanna Fernandes, Katarina Savel, Ankita Shah, Ravindra Agrawal, Anant Bhan, Abhijit Nadkarni, Akshita Sharma, Azaz Khan, Anuja Lahiri, Deepak Tugnawat, Neal Lesh, Vikram Patel and John Naslund

The article has been updated.
