# Supplementary figures and images for: Scaling up the task-sharing of psychological therapies: A formative study of the PEERS smartphone application for supervision and quality assurance in rural India
Source: Glob Ment Health (Camb). 2024 Feb 5;11:e20. doi: 10.1017/gmh.2024.11 (PMC10988170; doi:10.1017/gmh.2024.11)

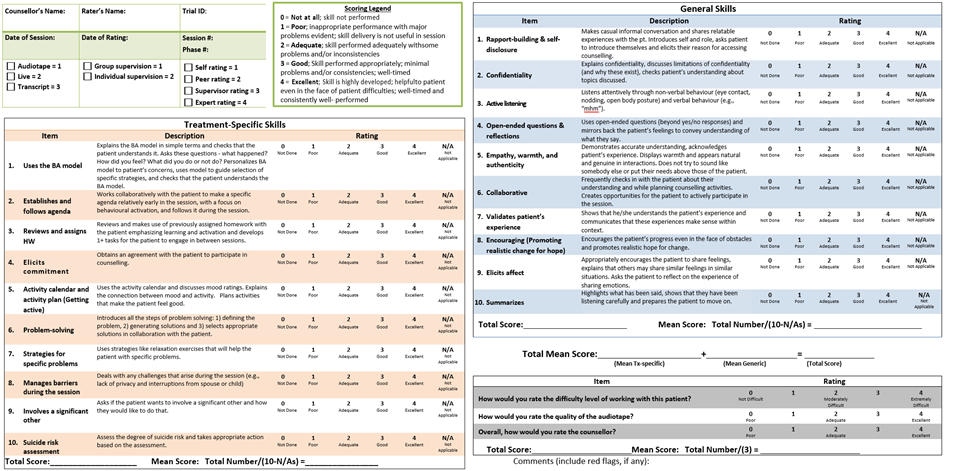

Supplement: Singla et al. supplementary material [file S2054425124000116sup001.png]
