# Supplementary material for: Scaling up the task-sharing of psychological therapies: A formative study of the PEERS smartphone application for supervision and quality assurance in rural India
Source: Glob Ment Health (Camb). 2024 Feb 5;11:e20. doi: 10.1017/gmh.2024.11 (PMC10988170; doi:10.1017/gmh.2024.11)
Supplement: Peer review history

# Editor report: Recommendation: Scaling up the task-sharing of psychological therapies: A formative study of the PEERS smartphone application for supervision and quality assurance in rural India — R0/PR2

**DOI:** 10.1017/gmh.2024.11.pr2

**Affiliations:** University of Pittsburgh, United States

Thank you for submitting this interesting manuscript which sought to explore the acceptability and feasibility of smartphone app for psychological treatment. 

The reviewers consider the manuscript relevant and an important addition to evidence on the subject. However, they have also shared some relevant feedback including comments and questions that I believe would strengthen the manuscript, especially the methods and results section.

I invite the authors to reflect and respond to the comments and suggestions of the reviewers and resubmit a revised version of the manuscript.

# Editor report: Decision: Scaling up the task-sharing of psychological therapies: A formative study of the PEERS smartphone application for supervision and quality assurance in rural India — R0/PR3

**DOI:** 10.1017/gmh.2024.11.pr3

**Affiliations:** London School of Hygiene & Tropical Medicine, United Kingdom of Great Britain and Northern Ireland

No accompanying comment.

# Editor report: Recommendation: Scaling up the task-sharing of psychological therapies: A formative study of the PEERS smartphone application for supervision and quality assurance in rural India — R1/PR5

**DOI:** 10.1017/gmh.2024.11.pr5

**Affiliations:** University of Pittsburgh, United States

No accompanying comment.

# Editor report: Decision: Scaling up the task-sharing of psychological therapies: A formative study of the PEERS smartphone application for supervision and quality assurance in rural India — R1/PR6

**DOI:** 10.1017/gmh.2024.11.pr6

**Affiliations:** London School of Hygiene & Tropical Medicine, United Kingdom of Great Britain and Northern Ireland

No accompanying comment.
